# An Oral Combination of Vitamins A, C, E, and Mg^++^ Improves Auditory Thresholds in Age-Related Hearing Loss

**DOI:** 10.3389/fnins.2018.00527

**Published:** 2018-07-31

**Authors:** Juan C. Alvarado, Verónica Fuentes-Santamaría, María C. Gabaldón-Ull, José M. Juiz

**Affiliations:** Instituto de Investigación en Discapacidades Neurológicas, Facultad de Medicina, Universidad de Castilla-La Mancha, Albacete, Spain

**Keywords:** micronutrients, vitamins, magnesium, presbycusis, aging, hearing loss, age-related hearing loss, auditory brainstem response

## Abstract

The increasing rate of age-related hearing loss (ARHL), with its subsequent reduction in quality of life and increase in health care costs, requires new therapeutic strategies to reduce and delay its impact. The goal of this study was to determine if ARHL could be reduced in a rat model by administering a combination of antioxidant vitamins A, C, and E acting as free radical scavengers along with Mg^++^, a known powerful cochlear vasodilator (ACEMg). Toward this goal, young adult, 3 month-old Wistar rats were divided into two groups: one was fed with a diet composed of regular chow (“normal diet,” ND); the other received a diet based on chow enriched in ACEMg (“enhanced diet,” ED). The ED feeding began 10 days before the noise stimulation. Auditory brainstem recordings (ABR) were performed at 0.5, 1, 2, 4, 8, 16, and 32 kHz at 3, 6–8, and 12–14 months of age. No differences were observed at 3 months of age, in both ND and ED animals. At 6–8 and 12–14 months of age there were significant increases in auditory thresholds and a reduction in the wave amplitudes at all frequencies tested, compatible with progressive development of ARHL. However, at 6–8 months threshold shifts in ED rats were significantly lower in low and medium frequencies, and wave amplitudes were significantly larger at all frequencies when compared to ND rats. In the oldest animals, differences in the threshold shift persisted, as well as in the amplitude of the wave II, suggesting a protective effect of ACEMg on auditory function during aging. These findings indicate that oral ACEMg may provide an effective adjuvant therapeutic intervention for the treatment of ARHL, delaying the progression of hearing impairment associated with age.

## Introduction

In the next three decades the number of people aged 60 or more will rise from 900 million to 2 billion, increasing in global rate from 12 to 22% (World Health Organization, 2017a). With the population growing older, an increase in pathologies related to aging is predicted. One is age-related hearing loss (ARHL) or presbycusis. ARHL currently affects about 24% of quadragenarian, 33% of sexagenarian and 66% of the septuagenarians in the world(Mathers et al., 2008; Ohlemiller and Frisina, 2008; Gopinath et al., 2009; Lin F.R. et al., 2011; Yamasoba et al., 2013). Associated to ARHL there is a significant reduction in the quality of life at an important humanitarian and socio-economic impact including health care costs (Huang and Tang, 2010; Ciorba et al., 2012; Kidd and Bao, 2012; World Health Organization, 2017b). Actually, ARHL is considered a major contributor to cognitive decline (Lin F.R. et al., 2011). To date there are no effective medications to cure or prevent ARHL. This is partly due to the fact that the etiopathogenesis of this sensory dysfunction is multifactorial and highly complex and still remains unclear (Huang and Tang, 2010; Fetoni et al., 2011; Yamasoba et al., 2013; Melgar-Rojas et al., 2015b), which limits therapeutic approaches.

Among mechanisms underlying ARHL (Chisolm et al., 2003; Mills and Schmiedt, 2004; Fetoni et al., 2011), one major contributor is oxidative stress (Fetoni et al., 2011; Fujimoto and Yamasoba, 2014; Alvarado et al., 2015b), i.e., an imbalance between removal and production of highly oxidative free radicals, by-products of oxidative metabolism. Accumulation of damage to the nuclear and mitochondrial genome and altered protein regulation and homeostasis, both at the core of the systemic aging process (López-Otín et al., 2013; Melgar-Rojas et al., 2015b), may lead to mitochondrial failure and breakdown of antioxidant enzymatic system with associated free radical build-up in the aging auditory receptor (Fetoni et al., 2011; Fujimoto and Yamasoba, 2014; Alvarado et al., 2015b). During aging, oxidative stress may easily target auditory hair cells, cells in the stria vascularis or spiral ganglion neurons because they are heavily dependent on oxidative metabolism, due to the unusually high metabolic demands of mechanoelectrical transduction. Excess free radicals engage in dysregulated redox reactions damaging membrane lipids and proteins essential for auditory signal transduction, ultimately leading to cell death and hearing loss (Ohlemiller, 2006; Chen et al., 2009; Bielefeld et al., 2010; Huang and Tang, 2010; Fetoni et al., 2011; Fujimoto and Yamasoba, 2014; Alvarado et al., 2015b). Excess free radical formation has also been associated with noise-induced hearing loss (NIHL) and drug-induced hearing loss (DIHL) (Henderson et al., 2006; Le Prell et al., 2007a,b; Bielefeld et al., 2010; Fetoni et al., 2011; Le Prell et al., 2014), suggesting that oxidative stress is a common pathogenic pathway in many pathologies affecting the auditory system (Alvarado et al., 2015b).

In addition to oxidative stress, alterations in the microculation of the stria vascularis, the structure responsible for the generation of the endocochlear potential (EP) needed for mechanotransduction, seem to influence ARHL. Age-dependent degeneration and atrophy of the stria vascularis, especially its microcirculation, seems a major contributor to ARHL (Schuknecht and Gacek, 1993; Gates et al., 2002), through profound impact in the maintenance of the EP, affecting hair cell activity and signal amplification (Mills and Schmiedt, 2004; Schmiedt, 2010; Shi, 2011; Lee, 2013). Actually, degenerative changes in cochlear microvasculature and/or reduction of blood flow associated with aging contribute to increased auditory thresholds (Mills and Schmiedt, 2004; Bielefeld et al., 2010; Fetoni et al., 2011; Alvarado et al., 2015b). In this regard, exposure to intense noise reduces the diameter of cochlear blood vessels, including those in the stria vascularis (Thorne and Nuttall, 1987; Shone et al., 1991; Fetoni et al., 2011; Le Prell et al., 2011a; Shi, 2011).

Considering the outlined mechanisms, experimental therapies combining antioxidant vitamins (Yamasoba et al., 1999; Yamashita et al., 2005; Le Prell et al., 2007a,b) and cochlear vasodilators (Cevette et al., 2003; Li et al., 2011), especially Mg^++^ (Le Prell et al., 2007a,b) have proven efficacy in improving auditory function after NIHL (Le Prell et al., 2007a,b) or DIHL (Le Prell et al., 2014). Likewise, as excess free radicals along with diminished cochlear blood flow share key roles in the pathophysiology of ARHL then, therapies targeting both excess free radicals and cochlear blood flow reduction may be a useful strategy to prevent onset and/or progression of ARHL. A potential use will require oral administration, so that demonstration of oral efficacy is relevant. Consequently, the goal of this study was to determine whether hearing loss could be reduced or delayed in an animal model of ARHL by administering an oral combination of antioxidant vitamins A, C, and E and Mg^++^ (ACEMg).

## Materials and Methods

### Animals

Data were obtained from 16 adult male Wistar rats (Charles River, Barcelona, Spain) that were housed in the animal facility at the Universidad de Castilla-La Mancha (Albacete, Spain). Upon arrival, animals were maintained on a 12/12-h light/dark cycle with free access to water and food. All procedures were approved by the Ethics Committee on Animal Experimentation at the University of Castilla-La Mancha (Permit Number: PR-2013-02-03) and conformed to Spanish (R.D. 53/2013; Law 32/2007) and European Union (Directive 2010/63/EU) regulations for the care and use of animals in research.

### Experimental Groups and Diet Supplement

Rats were divided into two groups: one was fed with a normal chow diet (“normal diet,” ND, *n* = 8), and the other was fed with a chow diet enriched in ACEMg (“enriched diet,” ED, *n* = 8) (Harlan Teklad Diet TD.110032) (Green et al., 2016). The ED consisted in a tocopherol-stripped soy-based diet supplemented with beta-carotene (vitamin A precursor, 1.05 g/kg), vitamin C (10.29 g/kg), vitamin E (7.76 g/kg) and magnesium (Mg, 13.48 g/kg) (Green et al., 2016). Feeding with ED began 10 days before the noise stimulation and was maintained until the end of the experiments.

### Noise Overstimulation

In order to accelerate presbycusis, rats in ND and ED groups (3 month-old) were exposed to a protocol of noise overstimulation that consisted of 1h continuous white noise at 118 dB SPL, 5 days per week (Alvarado et al., 2015a), until the animals reached an age of 12–14 months. The sound was delivered inside a methacrylate reverberating chamber with 60 × 70 × 40 (length × width × height) cm in the bottom part, and with tilted and non-parallel walls to avoid standing waves and ensure a more homogeneous sound field. The chamber was placed into a double wall sound–attenuating booth located inside a sound–attenuating room. ABR recordings were performed in both groups of animals at three different time points: 3 (ND3 and ED3), 6–8 (ND6 and ED6), and 12–14 (ND12 and ED12) months of age, based on a previous study of aging in Wistar rats (Alvarado et al., 2014).

### Auditory Brainstem Response (ABR) Recordings

Recordings were performed as previously described (Alvarado et al., 2012, 2014, 2016; Fuentes-Santamaría et al., 2012, 2013, 2014; Melgar-Rojas et al., 2015a). Briefly, rats were placed in a sound-attenuating, electrically shielded booth (EYMASA/INCOTRON S.L., Barcelona, Spain) located inside a sound-attenuating room. They were then anesthetized with isoflurane (1 L/min O_2_ flow rate) at 4% for induction and 2% for maintenance. Subdermal needle electrodes (Rochester Electro-Medical, Tampa, FL, United States) were placed at the vertex (non-inverting), in the right (inverting) and in the left (ground) mastoids. The temperature was monitored, using a non-electrical heating pad, with a rectal probe and maintained at 37.5 ± 1°C. For the stimulation and recording a BioSig System III (Tucker-Davis Technologies, Alachua, FL, United States) was used. ABRs were obtained stimulating with pure tone burst sounds (5 ms rise/fall time without a plateau with a cos2 envelope delivered at 20/s), at the following frequencies: 0.5, 1, 2, 4, 8, 16, and 32 kHz. The stimuli, generated digitally with SigGenRP software (Tucker-Davis Technologies, Alachua, FL, United States), were delivered into the external auditory meatus of the right ear through an EC-1 electrostatic speaker (Tucker-Davis Technologies), connected to an EDC1 electrostatic speaker driver (Tucker-Davis Technologies), using the RX6 Piranha Multifunction Processor hardware (Tucker-Davis Technologies, Alachua, FL, United States). Before the experiments, stimuli were calibrated using SigCal software (Tucker-Davis Technologies) and an ER-10B+ low noise microphone system (Etymotic Research Inc., Elk, Groove, IL, United States). The responses were filtered (0.3 – 3.0 kHz), averaged (500 waveforms) and stored for off-line analysis.

### ABR Parameters

Measures of all ABR parameters evaluated in the present study were performed in all frequencies tested. Values obtained in 3 month-old rats were used as a control in either group.

### Auditory Thresholds Analysis

Auditory threshold in ND and ED rats was defined as the stimulus intensity in dB that evoked peak–to–peak waves amplitudes greater than two standard deviations (SD) from background activity (Cediel et al., 2006; Garcia-Pino et al., 2009; Alvarado et al., 2012, 2014, 2016; Fuentes-Santamaría et al., 2012, 2013, 2014, 2017; Melgar-Rojas et al., 2015a). Evoked responses were recorded from 80 dB sound pressure level (SPL) in 5 dB descending steps, while background activity was defined as the basal activity recorded prior stimulus onset. During recordings, the maximum level of intensity was set at 80 dB to minimize any possible additional noise overstimulation (Gourévitch et al., 2009; Alvarado et al., 2012, 2014, 2016; Fuentes-Santamaría et al., 2012, 2013, 2014, 2017; Melgar-Rojas et al., 2015a). For statistical purposes, in any frequency where no auditory evoked responses were obtained at 80 dB, the auditory threshold was set at that intensity level (Subramaniam et al., 1992; Trowe et al., 2008; Alvarado et al., 2012, 2014, 2016; Fuentes-Santamaría et al., 2012, 2013, 2014, 2017; Melgar-Rojas et al., 2015a).

The threshold shift in both ND and ED animals was determined as the differences between the time points: 6–8M and 12–14M, minus the auditory thresholds at 3M (Alvarado et al., 2012, 2014, 2016; Fuentes-Santamaría et al., 2012, 2013, 2014; Melgar-Rojas et al., 2015a).

The percentage of variation of the threshold shift was calculated using the following formula (Meredith and Stein, 1983; Alvarado et al., 2007a,b, 2009, 2016; Fuentes-Santamaría et al., 2017):

%of variation=[(ATTP−ATCC)/(ATCC)]×100

Where ATTP is the auditory threshold in the time-points 6–8M and 12–14M, and ATCC is the auditory threshold at 3M (control condition).

### Wave Amplitude Analysis

The wave amplitude was calculated as the sum of the absolute values of the positive peak and the following negative wave trough (Popelar et al., 2008; Church et al., 2010, 2012b; Alvarado et al., 2012, 2014, 2016). This parameter was measured in the largest and the most consistent waves of the ABR in Wistar rats, waves I, II and IV (Church et al., 2010, 2012b; Alvarado et al., 2012, 2014, 2016; Melgar-Rojas et al., 2015a).

Wave amplitudes were normalized using the wave amplitudes ratio (Boettcher et al., 1996), that was calculated as follows:

Ratio=(WATP/WACC)

Where WATP is the amplitude of the waves in the time-points 6–8M and 12–14M, and WACC is the wave amplitudes at 3M.

The percentage of variation of the wave amplitude also was calculated using the following formula (Meredith and Stein, 1983; Alvarado et al., 2007a,b, 2009, 2016; Fuentes-Santamaría et al., 2017):

%of variation=[(WATP−WACC)/(WACC)]×100

Where WATP is the amplitude of the waves in the time-points 6–8M and 12–14M, and WACC is the wave amplitudes at 3M.

### Wave Latencies Analysis

Absolute and interpeak wave latencies were measured, as described elsewhere (Chiappa et al., 1979; Chen and Chen, 1991; Gourévitch et al., 2009; Alvarado et al., 2012, 2014, 2016). The positive absolute latency (PAL) was the time in milliseconds (ms) between the stimulus onset and the corresponding positive peak, and the negative absolute latency (NAL) the time in ms between the stimulus onset and the negative trough (Chiappa et al., 1979; Chen and Chen, 1991; Gourévitch et al., 2009; Alvarado et al., 2012, 2014). In the absolute latencies, 0.5 ms were added as part of the acoustic transit time, between the speaker’s diaphragm and the rat’s tympanic membrane. The positive and negative interpeak latencies were the intervals in ms between I–II, II–IV, and I–IV waveforms positive and negative components respectively.

### Statistical Analysis

Data in the present study are expressed as means ± SEM. All the measurements of the wave parameters were done at 80 dB SPL. Statistical comparisons among groups were performed using two-way repeated measures analysis of variance (ANOVA) with diet (normal diet vs. enhanced diet) as an independent variable and age (3M, 6–8M, and 12–14M) as a repeated independent variable. The dependent variables were all ABR parameters measured in the present manuscript. For each one of the frequency studied, it was evaluated the possible statistically significant main effect of the diet and the age. If the main analysis indicated a significant effect of one factor or an interaction between factors, a Scheffé *post hoc* analysis was made. Significance levels (α) and power (β) were set to 0.05 and 95%, respectively.

## Results

### Auditory Thresholds

**FIGURE 1:**
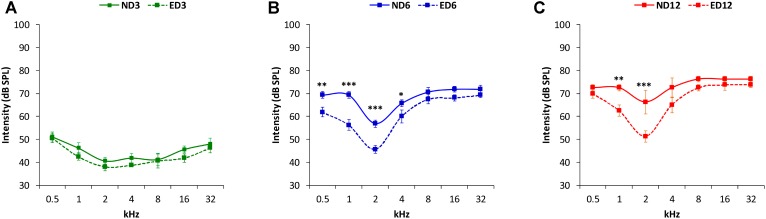
Line graphs illustrating auditory thresholds at different frequencies in ND and ED rats at 3 **(A)**, 6–8 **(B)**, and 12–14 **(C)** months of age. In both ND and ED animals, mean threshold values rose as the age of the animals increased **(B,C)**. However, at lower and medium frequencies the mean auditory thresholds in the ED6 **(B)** and ED12 **(C)** groups, were lower, closer to normal thresholds, than those observed in the ND group.

**FIGURE 2:**
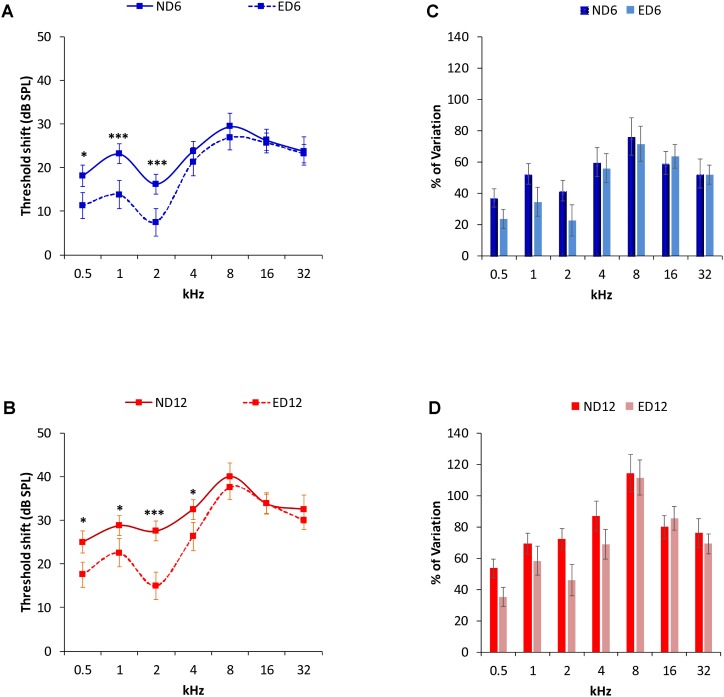
Auditory threshold shifts (line graphs) and percentage of variation (bar graphs) between ND and ED rats at 6–8 **(A,C)** and 12–14 months **(B,D)**. At 6–8 **(A)** and at 12–14 **(B)** months of age, threshold shifts in ED animals were smaller than those in ND animals in the lower and medium frequencies. No significant differences were observed at higher frequencies. The percentage of variation of the threshold shifts at 6–8 months of age **(C)**, were smaller in ED rats compared to those in ND rats, being 13.35, 17.74, and 18.77%, at 0.5, 1, and 2 kHz respectively. In the remaining frequencies, differences ranged from –4.64 to 4.28% **(C)**. In 12–14 month-old animals **(D)**, the percentage of variation of the threshold shifts in ED rats were smaller than those in ND rats. Percentages were 18.43, 11.08, 26.19, and 18.13% at 0.5, 1, 2, and 4 kHz; respectively, while at higher frequencies the differences ranged from –6.94 to 5.56%. ^∗^*p* < 0.05, ^∗∗∗^*p* < 0.001.

Analysis of the ABR recordings in 3 month-old rats showed that auditory thresholds obtained from animals assigned to ND and ED groups (**Figure 1A**) were comparable to those reported previously for Wistar rats (Jamesdaniel et al., 2009; Church et al., 2010; Alvarado et al., 2012, 2014, 2016; Pilati et al., 2012; Melgar-Rojas et al., 2015a). By 6–8 months of age in ND as well as in ED animals (**Figure 1B**), there were significant increases in the mean values of the auditory thresholds at all frequencies tested, as compared to those observed at 3 months (**Figure 1B**). Increases were even higher at 12–14 months of age, as shown in **Figure 1C**. Further analysis revealed that the threshold shift in ED animals, when compared to ND animals, at 6–8 months (**Figure 2A**) and at 12–14 months (**Figure 2B**) was significantly smaller, from an statistical standpoint, in the lower and medium frequencies. No statistically significant differences were detected in higher frequencies. Accordingly, the percentage of variation of the threshold shift at 6–8 months of age revealed that at 0.5, 1, and 2 kHz, increases in auditory thresholds were smaller (−13.35%, −17.74%, and −18.77%; respectively) in ED rats that in ND rats, whereas in the remaining frequencies, differences ranged from −4.64 to 4.28% (**Figure 2C**). Similarly, in 12–14-month-old animals the percentage of variation of the threshold shift was smaller in the ED group at 0.5 kHz (−18.43%), 1 kHz (−11.08%), 2 kHz (−26.19%) and 4 kHz (−18.13%) compared to ND animals, whereas in the higher frequencies, differences ranged from −6.94 to 5.56% (**Figure 2D**). ANOVA demonstrated a significant effect of ACEMg and age on auditory thresholds (*F*_2,274_ = 4.80, *p* < 0.05) and threshold shift (*F*_1,164_ = 3.68, *p* < 0.05). Scheffé *post hoc* test confirmed the significantly smaller auditory thresholds (**Figure 1**) and threshold shift values (**Figure 2**) in the lower and medium frequencies in ED rats at 6–8 months and at 12–14 months compared to ND rats.

### Waveform Amplitudes

**FIGURE 3:**
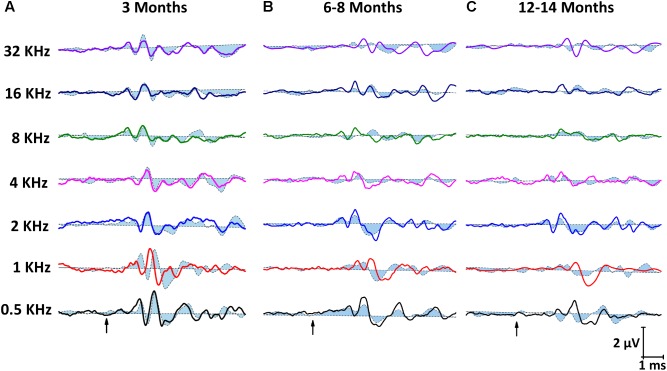
Line graphs showing examples of ABR recordings from ND (closed dashed lines) and ED (open solid lines) rats at all ages evaluated. In ND and ED rats at 3 months of age **(A)**, traces showed the characteristic 4 to 5 evoked waves after stimulus onset. No differences were apparent between groups. At 6–8 months of age **(B)**, there was a reduction in the amplitude of all waves at all frequencies, although it was more evident in ND rats. At 12–14 month of age **(C)**, despite the fact that reduction in the wave amplitudes was even more pronounced than that seen at 6–8 months, amplitudes in ND animals were still smaller than in ED rats. Arrows indicate stimulus onset.

Representative ABR recordings of ND rats (dashed closed traces) and ED rats (solid open traces) at 3 months of age are depicted in **Figure 3A**. As described elsewhere for the Wistar rat (Overbeck and Church, 1992; Church et al., 2010, 2012a,b; Alvarado et al., 2012, 2014, 2016), normal traces comprise four to five evoked waves, the largest being waves II, I, and IV (**Figure 3A**). However, at 6–8 months (**Figure 3B**), there was a decrease in the amplitude of all waves at all frequencies evaluated. This was more in ND rats, whereas ED rats showed smaller decreases in wave amplitudes. In 12–14 month-old animals (**Figure 3C**), the decrease in the amplitude of the waves was more pronounced than that observed at 6–8 months, and was still more evident in ND rats.

**FIGURE 4:**
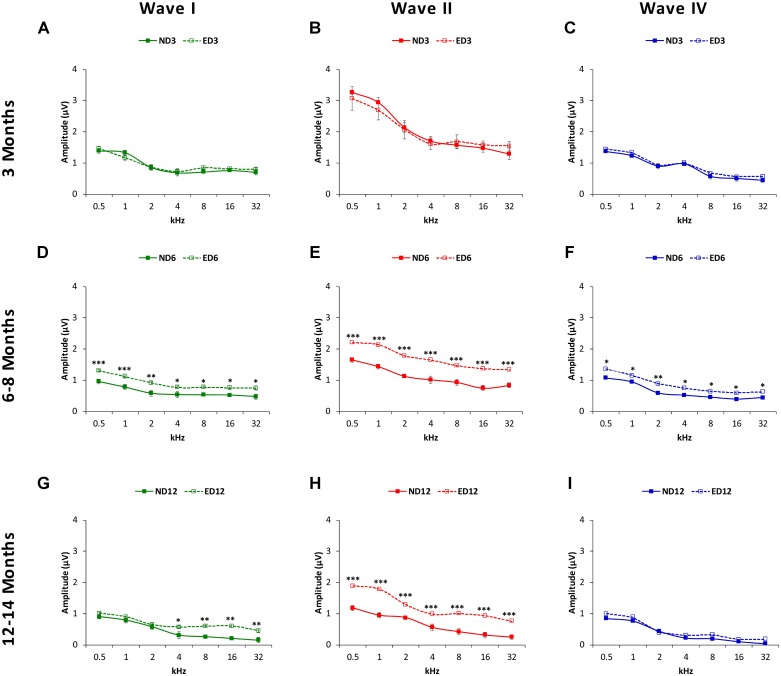
Line graphs depicting wave amplitudes (in μV) plotted as a function of frequency in ND (solid lines) and ED (dashed lines) rats at the different ages evaluated. At 3 months of age **(A–C)**, the mean values of the largest waves (I, II, and IV) were similar in both groups, with larger wave amplitudes in the lower frequencies and smaller at medium and higher frequencies, being wave II the largest of all. In 6–8-month-old rats **(D–F)**, the mean amplitudes of all waves were reduced, but values in ED rats were significantly larger compared to ND rats. At 12–14 months **(G–I)**, while the reduction in the mean amplitudes of all waves persisted, in the ED group values of wave II at all frequencies **(H)** and of wave I at higher frequencies **(G)** were still larger than those observed in ND animals. No differences were observed in wave IV **(I)**. ^∗^*p* < 0.05, ^∗∗^*p* < 0.01, ^∗∗∗^*p* < 0.001.

Measurements of the amplitude of waves I, II, and IV (see Materials and Methods) confirmed visual evaluation. At 3 months of age, wave amplitudes showed similar average values in ND and ED rats (**Figures 4A–C**). Both at 6–8 months (**Figures 4D–F**) and at 12–14 months (**Figures 4G–I**) there was a reduction in all wave amplitudes in both ND and ED animals, although ND rats had significantly lower mean amplitudes. ANOVA, confirmed statistically significant differences in the mean amplitudes of waves I (*F*_2,274_ = 5.56, *p* < 0.01), II (*F*_2,274_ = 8.20, *p* < 0.001) and IV (*F*_2,274_ = 3.59, *p* < 0.05) between ND and ED animals. According to Scheffé post-hoc test, the amplitude of waves I (**Figure 4D**), II (**Figure 4E**), and IV (**Figure 4F**) were significantly larger at 6–8 months of age in ED rats relative to those in ND rats at all frequencies studied. At 12–14 months, mean amplitude differences persisted in wave II (**Figure 4H**) at all frequencies, whereas in wave I (**Figure 4G**) they were only detected at the higher frequencies and no differences were observed in wave IV (**Figure 4I**).

**FIGURE 5:**
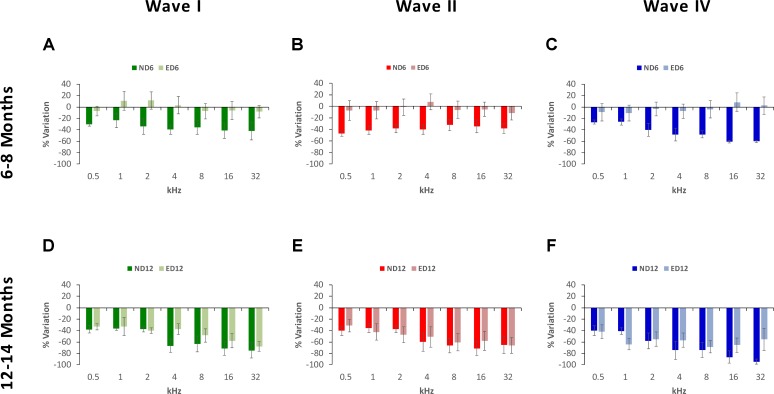
Bar graphs illustrating the percentage of variation in the wave amplitudes in older rats relative to the control condition (3-month-old rats). At 6–8 months of age, the percentage of variation in the wave amplitudes in ND rats was greater than in ED rats. These values in ND rats ranged from –23.48 to –42.11% for wave I **(A)**, from –32.00% to –47.07% for wave II **(B)** and from –26.31 to 60.51% for wave IV **(C)**, while in ED rats they ranged from –8.36 to 11.44% for wave I **(A)**, from –11.22 to 7.81% for wave II **(B)**, and from –10.30 to 8.60% for wave IV **(C)**. At 12–14 months of age, while variations relative to controls were larger, differences between both groups were reduced. In ND rats, these values fluctuated from –36.52 to –71.58% for wave I **(D)**, from –35.71 to –71.19% for wave II **(E)** and from –39.91 to 94.30% for wave IV **(F)**. Meanwhile, in ED rats, they ranged from –32.67 to 67.84% for wave I **(D)**, from 31.46 to 65.95% for wave II **(E)** and from –41.78 to 68.40% for wave IV **(F)**.

**FIGURE 6:**
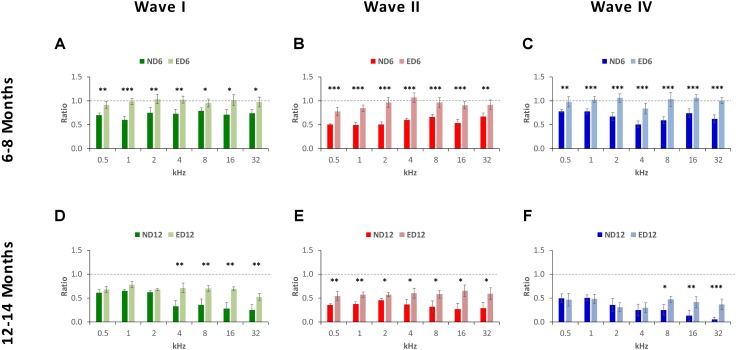
Bar graphs illustrating the wave amplitude ratio in older animals relative to the control condition. In ND rats at 6–8 months of age, the wave amplitude ratios for waves I **(A)**, II **(B)**, and IV **(C)** were smaller at all frequencies when compared to ED rats. In ND rats at 12–14 months of age, compared to ED rats, ratios were still smaller for wave II **(E)** at all frequencies assessed and for waves I **(D)** and for wave IV **(F)** at the higher frequencies. ^∗^*p* < 0.05, ^∗∗^*p* < 0.01, ^∗∗∗^*p* < 0.001.

The percentage of variation, relative to the control condition (3-month-old rats), revealed the magnitude of changes in wave amplitudes. As shown in **Figure 5**, variations at 6–8 months of age were as follows: in wave I from −23.48 to −42.11% in ND rats and from −8.36 to 11.44% in ED rats (**Figure 5A**); in wave II from −32.00 to −47.07% in ND animals and from −11.22 to 7.81% in ED animals (**Figure 5B**); and in wave IV from −26.31 to 60.51% in ND rats and from −10.30 to 8.60% in ED rats (**Figure 5C**). As rats grow older (12–14 months), these variations were larger and the differences between groups were reduced. For instance, in wave I the percentage of variation ranged from −36.52 to −71.58% in ND rats and from −32.67 to 67.84% in ED rats (**Figure 5D**); in wave II it ranged from −35.71 to −71.19% in ND rats and from −31.46 to 65.95% in ED rats (**Figure 5E**); and in wave IV variations ranged from −39.91 to 94.30% in ND rats and from −41.78 to 68.40% in ED rats (**Figure 5F**). ANOVA, after normalization of amplitude values of waves I (*F*_1,164_ = 3.07, *p* < 0.05), II (*F*_1,164_ = 4.45, *p* < 0.05) and IV (*F*_1,164_ = 3.41, *p* < 0.05) by using the wave amplitude ratio (see Materials and Methods), followed by Scheffé’s *post hoc* test, demonstrated that in the ND6 group ratios were always smaller in all waves and at all frequencies than those found in the ED6 group (**Figures 6A–C**). The same analysis performed in 12–14-month-old rats, revealed significant differences in the wave amplitude ratios, being smaller in wave II (**Figure 6E**) at all frequencies, and in waves I (**Figure 6D**) and IV (**Figure 6F**) at the higher frequencies in ND rats compared to ED rats.

### Waveform Absolute and Interpeak Latencies

**FIGURE 7:**
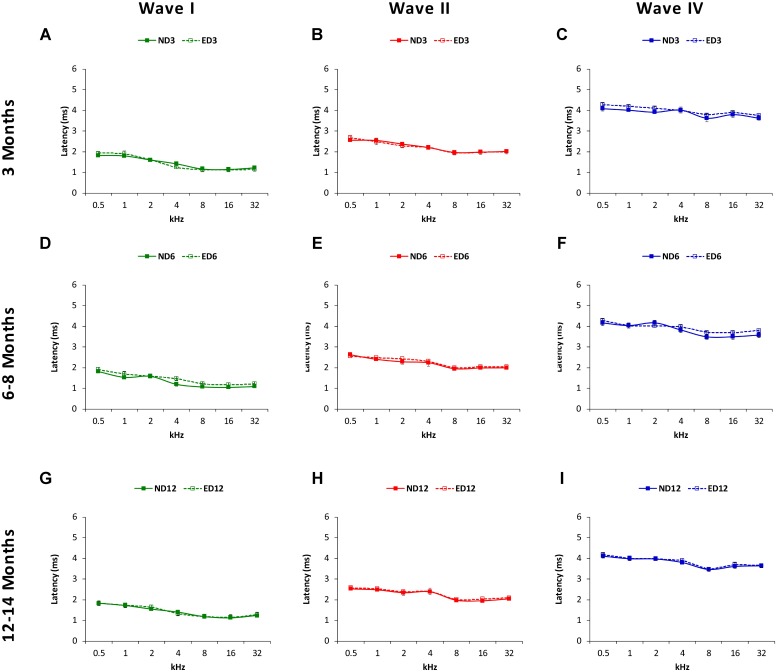
Line graphs illustrating absolute latencies of the positive peaks (ms) of waves I, II and IV plotted as a function of frequency in ND and ED rats. At 3 months of age **(A–C)**, the mean values for the absolute positive latencies were similar between ND and ED rats. At 6–8 months of age **(D–F)**, absolute latency times were longer, compared to 3 month-old rats. However, there were no differences between ND and ED rats in any wave at any frequency assessed. Similarly, although the absolute latency times were even longer at 12–14 months of age **(G–I)**, still no differences were observed in any wave at any frequency in the positive latency times when ND and ED rats were compared.

**FIGURE 8:**
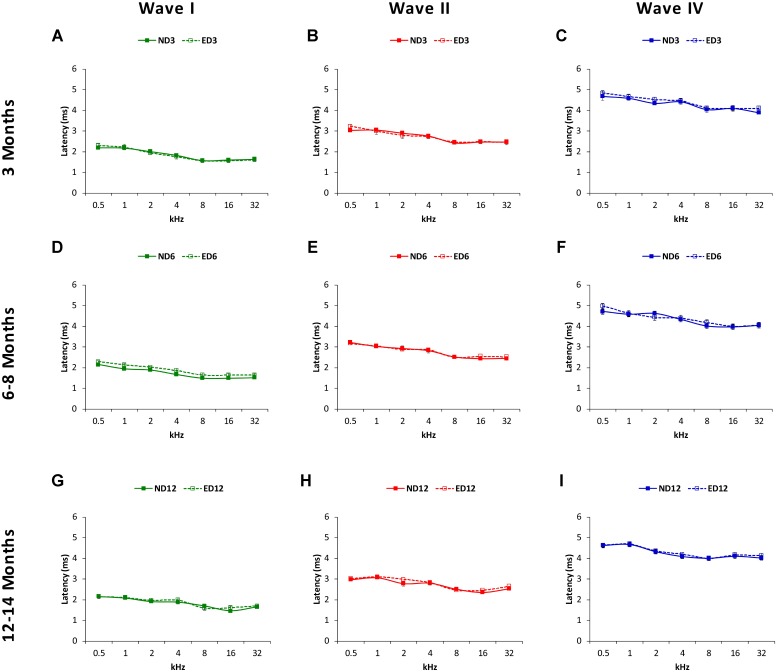
Line graphs illustrating absolute negative latencies (in ms) of waves I, II, and IV plotted as a function of frequency in ND and ED rats. The mean values for the absolute negative latencies in ND3 and ED3 rats **(A–C)** did not differ signficantly between them. Negative absolute latency times were longer at 6–8 months of age **(D–F)**, but there were no differences between ND6 and ED6 animals in any wave at any frequency assessed. Despite of the longer absolute negative latency times at 12–14 months of age **(G–I)**, still no differences were observed in any wave at any frequency when ND12 and ED12 rats where compared.

The mean values for the absolute positive and negative latencies are shown in **Figures 7**, **8**; respectively. As it can be observed, there were little or no differences between ND and ED animals at any age or at any frequency evaluated. At 3 months of age, the mean values found in both groups for the absolute positive (**Figures 7A–C**) as well as the negative latencies (**Figures 8A–C**) in all waves were similar to those described elsewhere for the Wistar rat (Jamesdaniel et al., 2009; Church et al., 2010; Alvarado et al., 2012, 2014, 2016; Pilati et al., 2012; Melgar-Rojas et al., 2015a). At 6–8 months of age, althought there were longer absolute latency times than those observed at 3 months of age, no aparent differences were observed, either in any wave or at any frequency, in the absolute positive (**Figures 7D–F**) and negative (**Figures 8D–F**) latency times of ND and ED rats. Likewise, at 12–14 months of age, in spite of the longer absolute latency times observed, the mean values of the absolute latency times, positive (**Figures 7G–I**) as well as negative (**Figures 8G–I**), were similar between ND and ED rats, in all waves and at all frequencies. ANOVA confirmed that there were no statistically significant differences in the mean values of the positive (wave I: *F*_2,270_ = 1.65, *p* > 0.05; wave II: *F*_2,270_ = 0.21, *p* > 0.05; wave IV: *F*_2,266_ = 0.52, *p* > 0.05; **Figure 7**) and negative (wave I: *F*_2,270_ = 1.57, *p* > 0.05; wave II: *F*_2,270_ = 0.03, *p* > 0.05; wave IV: *F*_2,266_ = 0.31, *p* > 0.05; **Figure 8**) absolute latency times.

**FIGURE 9:**
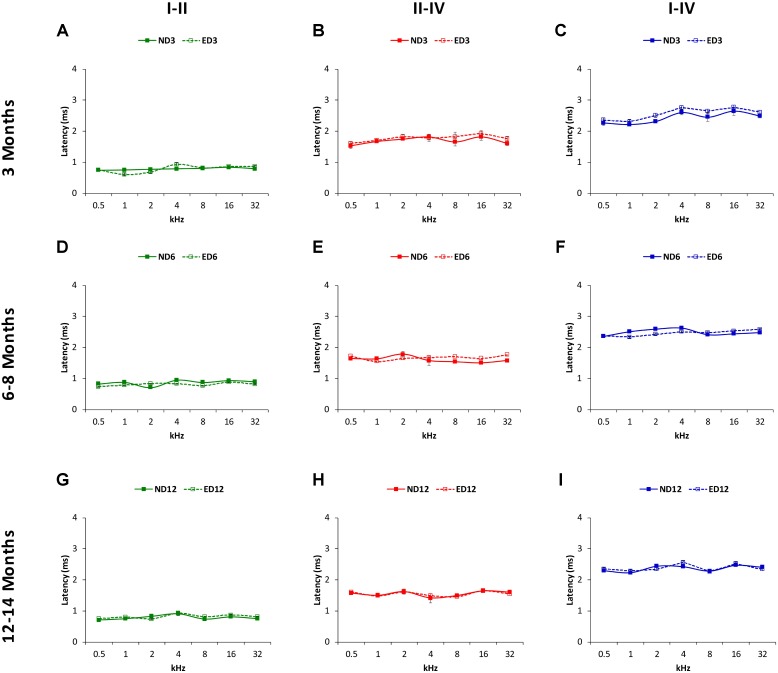
Line graphs illustrating interpeak positive latencies (in ms) plotted as a function of frequency in ND and ED rats. Regardless the age of the animal, the mean values of the interpeak positive latencies observed at 3 **(A–C)**, 6–8 **(D–F)**, and 12–14 **(G–I)** months were similar in ND and ED rats at all frequencies evaluated.

**FIGURE 10:**
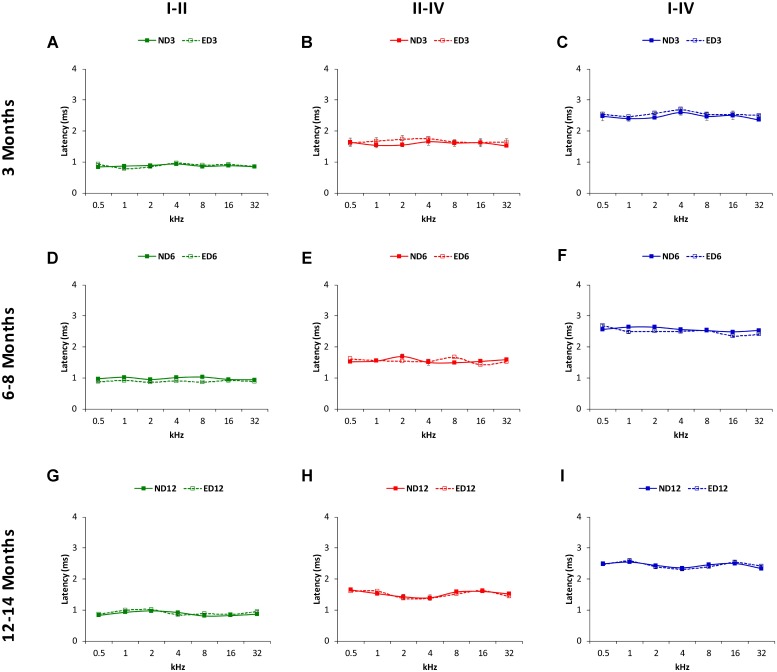
Line graphs illustrating interpeak negative latencies (in ms) plotted as a function of frequency in ND and ED rats. Similar to interpeak positive latencies, no significant differences were observed at any stimulus frequency, when ND3 vs. ED3 **(A–C)**, ND6 vs. ED6 **(D–F)** and ND12 vs. ED12 **(G–I)** were compared.

Regarding the positive (**Figure 9**) and negative (**Figure 10**) interpeak latencies, as described for the absolute latencies, no apparent differences were observed at any age or at any frequency when ND and ED rats were compared. In ND3 and ED3 animals, the mean values of the positive (**Figures 9A–C**) and negative (**Figures 10A–C**) interpeak latencies were similar to those described previously for the Wistar rat (Jamesdaniel et al., 2009; Church et al., 2010; Alvarado et al., 2012, 2014, 2016; Pilati et al., 2012; Melgar-Rojas et al., 2015a). In ND6 and ED6 rats, positive (**Figures 9D–F**) and negative (**Figures 10D–F**) interpeak latencies mean values were not different either between them or when compared to the values observed in 3 month-old animals. In ND12 and ED12 rats, positive (**Figures 9G–I**) and negative (**Figures 10G–I**) interpeak latencies mean values also were similar between them and when compared to younger rats. ANOVA demonstrated the absence of statistically significant differences in the mean values of the positive (I–II: F_2,270_ = 1.90, *p* > 0.05; II–IV: *F*_2,270_ = 1.44, *p* > 0.05; I–IV: *F*_2,266_ = 1.03, *p* > 0.05; **Figure 9**) and negative (I–II: F_2,270_ = 1.45, *p* > 0.05; II–IV: *F*_2,270_ = 1.07, *p* > 0.05; I–IV: *F*_2,266_ = 1.72, *p* > 0.05; **Figure 10**) interpeak latency times.

## Discussion

The present study demonstrates a protective effect of oral ACEMg in ARHL, as seen in changes in auditory thresholds and wave amplitudes of ABR recordings in rats fed with ACEMg-enriched chow (ED rats) compared to control rats fed with regular chow (ND rats). In the ACEMg-supplemented rats, the mean threshold shifts at 6–8 and 12–14 months of age were significantly reduced in the lower and medium frequencies when compared to those in ND rats. Additionally, the mean wave amplitudes in the three largest waves were significantly larger at 6–8 months at all frequencies evaluated. At 12–14 months, in ED animals, the mean amplitude of wave II was significantly larger at all frequencies, and that of waves I and IV at the higher frequencies, compared to the ND rats. The present findings suggest that oral therapies targeting excess of free radicals and reduced cochlear blood flow may improve auditory function during aging in Wistar rats, a validated model of ARHL (Alvarado et al., 2014).

With the continuous improvement in the quality of life and health care systems, the number of people reaching the sixties or older ages will rise from 12 to 22% in the whole world in about 30 years (World Health Organization, 2017a). As a consequence, there will be an increase in age-related pathologies such as ARHL, which represents the most frequent sensory disability in aged people (Van Eyken et al., 2007; Bielefeld et al., 2010; Huang and Tang, 2010). Despite of its high prevalence and the wealth of studies available, the complex etiopathogenesis of this chronic, continuous and irreversible condition, is still not well understood (Huang and Tang, 2010; Fetoni et al., 2011; Yamasoba et al., 2013; Tavanai and Mohammadkhani, 2017). Based on evaluations of the temporal bones and audiometric tests of aged patients, six histopathological types of presbycusis have been described: (1) sensory, which occurs with loss of outer hair cells and supporting cells; (2) neural, which is associated with loss of spiral ganglion neurons; (3) strial or metabolic, related to degeneration or atrophy of the stria vascularis; (4) cochlear conductive or mechanical, which is associated with changes in the stiffness of the basilar membrane; (5) mixed presbycusis, characterized by the coexistence of more than one of those forms of presbycusis; and (6) indeterminate presbycusis, represented by the cases (one in four people) that do not fit into any of the types above mentioned and therefore, its pathophysiological mechanism is unknown (Schuknecht et al., 1974; Schuknecht and Gacek, 1993; Bielefeld et al., 2010; Huang and Tang, 2010; Schmiedt, 2010; Lee, 2013; Yamasoba et al., 2013; Melgar-Rojas et al., 2015b). This clear-cut classification has been challenged (Ohlemiller and Frisina, 2008; Melgar-Rojas et al., 2015b) as it is likely that in most instances clinical presbycusis comprises an accumulation of cochlear pathologies (Engle et al., 2013). However, it is still valid as a framework to add new knowledge on ARHL mechanisms. In fact, several interrelated factors have been postulated to explain the pathophysiology of this sensory dysfunction (Bielefeld et al., 2010; Huang and Tang, 2010; Schmiedt, 2010; Fetoni et al., 2011; Yamasoba et al., 2013; Melgar-Rojas et al., 2015b). Oxidative stress and degenerative structural and functional alterations in the stria vascularis, likely in combination in most instances, seem to have a relevant role in the genesis of ARHL. As far as oxidative stress is concerned, ARHL reflects incapacity of the endogenous antioxidant systems to eliminate excess free radicals. This, in turn, would induce oxidative damage and loss of hair cells, supporting cells, ganglion cells, and fibrocytes in the stria vascularis (Bielefeld et al., 2010; Huang and Tang, 2010; Fetoni et al., 2011; Yamasoba et al., 2013; Fujimoto and Yamasoba, 2014; Alvarado et al., 2015b; Melgar-Rojas et al., 2015b). Excess free radical formation seems to be part of a common pathogenic pathway (Alvarado et al., 2015b; Tavanai and Mohammadkhani, 2017) which is involved in other forms of hearing loss such as NIHL and DIHL (Henderson et al., 2006; Le Prell et al., 2007a,b; Bielefeld et al., 2010; Fetoni et al., 2011) as weel as in many neurodegenerative pathologies (Ames et al., 1993; Lin and Beal, 2006; Gardiner et al., 2009) that present high incidence of hearing loss (Lin Y.S. et al., 2011; Vitale et al., 2012; Hung et al., 2015; Hardy et al., 2016; Folmer et al., 2017; Profant et al., 2017; Zheng et al., 2017). Age-related degeneration or atrophy of the stria vascularis, including the accompanying microvasculature, affects the EP and therefore, the amplification of acoustic signals, leading to an increase in auditory thresholds (Mills and Schmiedt, 2004; Bielefeld et al., 2010; Huang and Tang, 2010; Schmiedt, 2010; Fetoni et al., 2011; Shi, 2011; Lee, 2013; Melgar-Rojas et al., 2015b). It has been proposed that the strial pathology is the primary cause of hearing loss in aged subjects (Schuknecht and Gacek, 1993; Gates et al., 2002; Schmiedt, 2010). Knowing the pathological cellular mechanisms involved in the genesis of the ARHL, will help to understand the signs and symptoms of this condition and therefore, to develop new treatment approaches.

To date, there are no therapeutic strategies to prevent or decrease the progression of presbycusis. In the absence of a cure, and given that ARHL it is not a preventable condition, treatments have focused mainly on reducing or avoiding any possible risk factors such as smoking, noise exposure, ototoxic drugs, alcohol, hormones, diet, which could accelerate its appearance (Van Eyken et al., 2007; Huang and Tang, 2010; Walling and Dickson, 2012). Attempts to restore hearing during aging are based on the use of gene and stem cells therapies (Revuelta et al., 2017). However, although results seem to be promising, they are still far from being applied to humans (Bielefeld et al., 2010; Huang and Tang, 2010; Fetoni et al., 2011; Walling and Dickson, 2012; Revuelta et al., 2017). Currently, it is likely that a promising therapeutic principle would be the use of substances that could target the above mentioned pathophysiological mechanisms. Accordingly, antioxidants have been used in experimental animal models and even in humans in attempts to reduce the impact of ARHL. Although some results apparently are contradictory (Fetoni et al., 2011; Sha et al., 2012), most agree in that antioxidants can slow or decrease the progression of hearing loss associated with aging (Seidman, 2000; Henderson et al., 2006; Heman-Ackah et al., 2010; Fetoni et al., 2011; Tavanai and Mohammadkhani, 2017). Consistent with this, our findings show that an oral combination of antioxidant vitamins (A, C, and E) to reduce oxidative stress by scavenging free radicals, plus the vasodilator Mg^++^, to increase strial blood flow, among other poorly understood mechanisms (Sendowski et al., 2011), produce a clear protective effect of auditory function in an animal model of ARHL. The threshold shifts observed in aged rats fed with chow enriched in ACEMg (ED) were significantly reduced at the lower and medium frequencies compared to those observed in age-matched rats fed with regular chow (ND), indicating an improvement in the auditory thresholds. Our data also show that the protective effect of ED on hearing was also reflected in the amplitudes of the waveforms. The decrease in the wave amplitudes observed in the ABR of aged ND rats at all frequencies evaluated, was significantly smaller in ED rats of the same age. The improvement in auditory thresholds and wave amplitudes in the ACEMg-supplemented animals reflects a more effective and adequate neuronal response to the auditory stimulus, than the one that they should have for their corresponding age. However, the finding that wave latencies are unaffected by ACEMg treatment suggests that there are central components of ARHL which are differentially sensitive to otoprotection. Therefore, these results suggest that oral administration of ACEMg, likely acting on free radicals and the stria vascularis delays the progression of hearing impairment associated with age in the Wistar rats.

Although this is the first time that an ACEMg-supplemented diet has been used for the treatment of ARHL, a similar combination of micronutrients has been successfully used, for the treatment of NIHL in pigmented guinea pigs (Le Prell et al., 2007a) and mice (Le Prell et al., 2011b), and for the treatment of gentamycin in pigmented guinea pigs (Le Prell et al., 2014). The effectiveness of oral administration of ACEMg in ARHL demonstrated here opens the route to facilitate easy administration in human applications. The fact that there is an improvement in auditory function in animal models of ARHL, NIHL and some forms of DIHL (reduction of the threshold shift) with the administration of ACEMg, lends further support to the idea of a common pathophysiological pathway among several conditions leading to hearing loss (Alvarado et al., 2015b; Tavanai and Mohammadkhani, 2017). Considering the multifactorial nature of the pathophysiological mechanisms involved in the genesis of presbycusis, it would be reasonable to expect that targeting more than one pathway and even different points in the same pathway, will have a greater impact over the progression of this sensory disability. For instance, the use of a combination of six antioxidants, acting in four different sites of the oxidative pathway, induce a threshold shift reduction in C57BL/6 mice, an animal model of ARHL (Heman-Ackah et al., 2010). However, the individual use of ACE or Mg in pigmented guinea pigs fails to produce a significant protection in auditory function (Le Prell et al., 2007a), suggesting the importance of the synergism and/or additivity of these micronutrients for the treatment of hearing loss. Even though antioxidant effects could overlap, it is known that the main effects of vitamin A are as scavenger of singlet oxygen, while vitamin C and vitamin E scavenge extracellular free radicals and membrane peroxyl radicals, respectively; therefore, contributing collectively to reduce the excess of free radicals in oxidative pathways (Le Prell et al., 2007a; Alvarado et al., 2015b). Additionally, it is worth noting that in the present study, the protective effect of ACEMg was not restricted to the auditory thresholds, as it was also evident in the wave amplitudes, suggesting an improvement in signal amplification. Accordingly, it might be expected that the vasodilator effect of Mg^++^ could restore at least partially, blood flow in the stria vascularis, reestablishing the EP and thus, improving acoustic signal amplification, which is reflected in higher amplitudes of the ABR waves in ED rats as compared to ND rats. Also, other potential mechanisms of Mg^++^ otoprotection related to calcium antagonism, antioxidation or NMDA receptor blockade (Sendowski et al., 2011) cannot be ruled out.

## Conclusion

We provide evidence that oral ACEMg preserves hearing in an animal model of accelerated ARHL, as shown by improved thresholds and amplitudes in ABR recordings. Future studies will be aimed at dissecting cellular mechanisms and improvement of ACEMg otoprotection in ARHL.

## Author Contributions

All authors had full access to all the data in the study and take responsibility for the integrity of the data and the accuracy of the data analysis. JA, VF-S, and JJ: study concept and design. JA, VF-S, MG-U: acquisition of data. JA and VF-S: statistical analysis and interpretation of the data. VF-S and JA: drafting of the manuscript. JA, VF-S, and JJ: critical revision of the manuscript for important intellectual content. JJ: obtaining funding.

## Conflict of Interest Statement

JJ, JA, and VF-S are co-inventors of the US Patents 9,889,156, “Method for treating noise-induced hearing loss (NIHL)” and 9,919,008, “Methods for treating age-related hearing loss (ARHL)”. Both patents are based on the use of oral ACEMg. The remaining author declares that the research was conducted in the absence of any commercial or financial relationships that could be construed as a potential conflict of interest.

## References

[B1] Alvarado J. C. Fuentes-Santamaría V. Gabaldón-Ull M. C. Blanco J. L. Juiz J. M. (2014). Wistar rats: a forgotten model of age-related hearing loss. *Front. Aging Neurosci.* 6:29. 10.3389/fnagi.2014.00029 24634657 PMC3942650

[B2] Alvarado J. C. Fuentes-Santamaría V. Gabaldón-Ull M. C. Jareño-Flores T. Miller J. M. Juiz J. M. (2016). Noise-induced “Toughening” effect in wistar rats: enhanced auditory brainstem responses are related to calretinin and nitric oxide synthase upregulation. *Front. Neuroanat.* 10:19 10.3389/fnana.2016.00019 PMC4815363 27065815

[B3] Alvarado J. C. Fuentes-Santamaría V. Jareño-Flores T. Blanco J. L. Juiz J. M. (2012). Normal variations in the morphology of auditory brainstem response (ABR) waveforms: a study in wistar rats. *Neurosci. Res.* 73 302–311. 10.1016/j.neures.2012.05.001 22595234

[B4] Alvarado J. C. Fuentes-Santamaría V. Melgar-Rojas P. Gabaldón-Ull M. C. Juiz J. M. (2015a). *Short-Duration and Long-Term Noise Overstimulation Could Increase Hearing Loss in Aging Wistar Rat. SENC Communications Abstracts Book.* Available at: https://www.congresomovil.com/senc2015

[B5] Alvarado J. C. Fuentes-Santamaría V. Melgar-Rojas P. Valero M. L. Gabaldón-Ull M. C. Miller J. M. (2015b). Synergistic effects of free radical scavengers and cochlear vasodilators: a new otoprotective strategy for age-related hearing loss. *Front. Aging Neurosci.* 7:86. 10.3389/fnagi.2015.00086 26029103 PMC4432684

[B6] Alvarado J. C. Stanford T. R. Rowland B. A. Vaughan J. W. Stein B. E. (2009). Multisensory integration in the superior colliculus requires synergy among corticocollicular inputs. *J. Neurosci.* 29 6580–6592. 10.1523/jneurosci.0525-09.2009 19458228 PMC2805025

[B7] Alvarado J. C. Stanford T. R. Vaughan J. W. Stein B. E. (2007a). Cortex mediates multisensory but not unisensory integration in superior colliculus. *J. Neurosci.* 27 12775–12786. 10.1523/jneurosci.3524-07.2007 18032649 PMC6673293

[B8] Alvarado J. C. Vaughan J. W. Stanford T. R. Stein B. E. (2007b). Multisensory versus unisensory integration: contrasting modes in the superior colliculus. *J. Neurophysiol.* 97 3193–3205. 10.1152/jn.00018.2007 17329632

[B9] Ames B. N. Shigenaga M. K. Hagen T. M. (1993). Oxidants, antioxidants, and the degenerative diseases of aging. *Proc. Natl. Acad. Sci. U.S.A.* 90 7915–7922. 10.1073/pnas.90.17.7915 8367443 PMC47258

[B10] Bielefeld E. C. Tanaka C. Chen G. Henderson D. (2010). Age-related hearing loss: Is it a preventable condition? *Hear. Res.* 264 98–107. 10.1016/j.heares.2009.09.001 19735708 PMC2868117

[B11] Boettcher F. A. Mills J. H. Swerdloff J. L. Holley B. L. (1996). Auditory evoked potentials in aged gerbils: responses elicited by noises separated by a silent gap. *Hear. Res.* 102 167–178. 10.1016/S0378-5955(96)90016-7 8951460

[B12] Cediel R. Riquelme R. Contreras J. Díaz A. Varela-Nieto I. (2006). Sensorineural hearing loss in insulin-like growth factor I-null mice: a new model of human deafness: Hearing loss in Igf-1-mutant mice. *Eur. J. Neurosci.* 23 587–590. 10.1111/j.1460-9568.2005.04584.x 16420467

[B13] Cevette M. J. Vormann J. Franz K. (2003). Magnesium and hearing. *J. Am. Acad. Audiol.* 14 202–212. 12940704

[B14] Chen G.-D. Li M. Tanaka C. Bielefeld E. C. Hu B.-H. Kermany M. H. (2009). Aging outer hair cells (OHCs) in the Fischer 344 rat cochlea: Function and morphology. *Hear. Res.* 248 39–47. 10.1016/j.heares.2008.11.010 19111601

[B15] Chen T. J. Chen S. S. (1991). Generator study of brainstem auditory evoked potentials by a radiofrequency lesion method in rats. *Exp. Brain Res.* 85 537–542. 10.1007/BF00231737 1915709

[B16] Chiappa K. H. Gladstone K. J. Young R. R. (1979). Brain stem auditory evoked responses: studies of waveform variations in 50 normal human subjects. *Arch. Neurol.* 36 81–87. 10.1001/archneur.1979.00500380051005 420627

[B17] Chisolm T. H. Willott J. F. Lister J. J. (2003). The aging auditory system: anatomic and physiologic changes and implications for rehabilitation. *Int. J. Audiol.* 42(Suppl. 2), S3–S10. 10.3109/14992020309074637 12918622

[B18] Church M. W. Adams B. R. Anumba J. I. Jackson D. A. Kruger M. L. Jen K.-L. (2012a). Repeated antenatal corticosteroid treatments adversely affect neural transmission time and auditory thresholds in laboratory rats. *Neurotoxicol. Teratol.* 34 196–205. 10.1016/j.ntt.2011.09.004 21963399 PMC3268869

[B19] Church M. W. Hotra J. W. Holmes P. A. Anumba J. I. Jackson D. A. Adams B. R. (2012b). Auditory brainstem response (ABR) abnormalities across the life span of rats prenatally exposed to alcohol. *Alcohol. Clin. Exp. Res.* 36 83–96. 10.1111/j.1530-0277.2011.01594.x 21815896 PMC3210930

[B20] Church M. W. Jen K.-L. C. Anumba J. I. Jackson D. A. Adams B. R. Hotra J. W. (2010). Excess omega-3 fatty acid consumption by mothers during pregnancy and lactation caused shorter life span and abnormal ABRs in old adult offspring. *Neurotoxicol. Teratol.* 32 171–181. 10.1016/j.ntt.2009.09.006 19818397 PMC2839050

[B21] Ciorba A. Bianchini C. Pelucchi S. Pastore A. (2012). The impact of hearing loss on the quality of life of elderly adults. *Clin. Interv. Aging* 7 159–163. 10.2147/CIA.S26059 22791988 PMC3393360

[B22] Engle J. R. Tinling S. Recanzone G. H. (2013). Age-related hearing loss in rhesus monkeys is correlated with cochlear histopathologies. *PLoS One* 8:e55092. 10.1371/journal.pone.0055092 23390514 PMC3563598

[B23] Fetoni A. R. Picciotti P. M. Paludetti G. Troiani D. (2011). Pathogenesis of presbycusis in animal models: a review. *Exp. Gerontol.* 46 413–425. 10.1016/j.exger.2010.12.003 21211561

[B24] Folmer R. L. Vachhani J. J. Theodoroff S. M. Ellinger R. Riggins A. (2017). Auditory processing abilities of Parkinson’s disease patients. *Biomed Res. Int.* 2017:2618587. 10.1155/2017/2618587 28546963 PMC5435898

[B25] Fuentes-Santamaría V. Alvarado J. C. Gabaldón-Ull M. C. Juiz J. M. (2013). Upregulation of insulin-like growth factor and interleukin 1β occurs in neurons but not in glial cells in the cochlear nucleus following cochlear ablation: upregulation of IGF-1 and IL-1β in Cochlear Nucleus. *J. Comp. Neurol.* 521 3478–3499. 10.1002/cne.23362 23681983

[B26] Fuentes-Santamaría V. Alvarado J. C. Juiz J. M. (2012). Long-term interaction between microglial cells and cochlear nucleus neurons after bilateral cochlear ablation. *J. Comp. Neurol.* 520 2974–2990. 10.1002/cne.23088 22351306

[B27] Fuentes-Santamaría V. Alvarado J. C. López-Muñoz D. F. Melgar-Rojas P. Gabaldón-Ull M. C. Juiz J. M. (2014). Glia-related mechanisms in the anteroventral cochlear nucleus of the adult rat in response to unilateral conductive hearing loss. *Front. Neurosci.* 8:319. 10.3389/fnins.2014.00319 25352772 PMC4195288

[B28] Fuentes-Santamaría V. Alvarado J. C. Melgar-Rojas P. Gabaldón-Ull M. C. Miller J. M. Juiz J. M. (2017). The role of glia in the peripheral and central auditory system following noise overexposure: contribution of TNF-α and IL-1β to the pathogenesis of hearing loss. *Front. Neuroanat.* 11:9. 10.3389/fnana.2017.00009 28280462 PMC5322242

[B29] Fujimoto C. Yamasoba T. (2014). Oxidative stresses and mitochondrial dysfunction in age-related hearing loss. *Oxid. Med. Cell. Longev.* 2014:582849. 10.1155/2014/582849 25110550 PMC4106174

[B30] Garcia-Pino E. Caminos E. Juiz J. M. (2009). KCNQ5 reaches synaptic endings in the auditory brainstem at hearing onset and targeting maintenance is activity-dependent. *J. Comp. Neurol.* 518 1301–1314. 10.1002/cne.22276 20151361

[B31] Gardiner J. Barton D. Overall R. Marc J. (2009). Neurotrophic support and oxidative stress: converging effects in the normal and diseased nervous system. *Neuroscientist* 15 47–61. 10.1177/1073858408325269 19218230

[B32] Gates G. A. Mills D. Nam B. D’Agostino R. Rubel E. W. (2002). Effects of age on the distortion product otoacoustic emission growth functions. *Hear. Res.* 163 53–60. 10.1016/S0378-5955(01)00377-X 11788199

[B33] Gopinath B. Rochtchina E. Wang J. J. Schneider J. Leeder S. R. Mitchell P. (2009). Prevalence of age-related hearing loss in older adults: blue mountains study. *Arch. Intern. Med.* 169 415–416. 10.1001/archinternmed.2008.597 19237727

[B34] Gourévitch B. Doisy T. Avillac M. Edeline J.-M. (2009). Follow-up of latency and threshold shifts of auditory brainstem responses after single and interrupted acoustic trauma in guinea pig. *Brain Res.* 1304 66–79. 10.1016/j.brainres.2009.09.041 19766602

[B35] Green K. L. Swiderski D. L. Prieskorn D. M. DeRemer S. J. Beyer L. A. Miller J. M. (2016). ACEMg diet supplement modifies progression of hereditary deafness. *Sci. Rep.* 6:22690. 10.1038/srep22690 26965868 PMC4786814

[B36] Hardy C. J. D. Marshall C. R. Golden H. L. Clark C. N. Mummery C. J. Griffiths T. D. (2016). Hearing and dementia. *J. Neurol.* 263 2339–2354. 10.1007/s00415-016-8208-y 27372450 PMC5065893

[B37] Heman-Ackah S. E. Juhn S. K. Huang T. C. Wiedmann T. S. (2010). A combination antioxidant therapy prevents age-related hearing loss in C57BL/6 mice. *Otolaryngol. Head Neck Surg.* 143 429–434. 10.1016/j.otohns.2010.04.266 20723783

[B38] Henderson D. Bielefeld E. C. Harris K. C. Hu B. H. (2006). The role of oxidative stress in noise-induced hearing loss. *Ear Hear* 27 1–19. 10.1097/01.aud.0000191942.36672.f3 16446561

[B39] Huang Q. Tang J. (2010). Age-related hearing loss or presbycusis. *Eur. Arch. Otorhinolaryngol.* 267 1179–1191. 10.1007/s00405-010-1270-7 20464410

[B40] Hung S.-C. Liao K.-F. Muo C.-H. Lai S.-W. Chang C.-W. Hung H.-C. (2015). Hearing loss is associated with risk of Alzheimer’s Disease: a case-control study in older people. *J. Epidemiol.* 25 517–521. 10.2188/jea.JE20140147 25986155 PMC4517989

[B41] Jamesdaniel S. Ding D. Kermany M. H. Jiang H. Salvi R. Coling D. (2009). Analysis of cochlear protein profiles of Wistar, Sprague-Dawley, and Fischer 344 Rats with normal hearing function. *J. Proteome Res.* 8 3520–3528. 10.1021/pr900222c 19432484

[B42] Kidd A. R. III Bao J. (2012). Recent advances in the study of age-related hearing loss: a mini-review. *Gerontology* 58 490–496. 10.1159/000338588 22710288 PMC3766364

[B43] Le Prell C. G. Dolan D. F. Bennett D. C. Boxer P. A. (2011a). Nutrient plasma levels achieved during treatment that reduces noise-induced hearing loss. *Transl. Res.* 158 54–70. 10.1016/j.trsl.2011.02.003 21708356 PMC3125531

[B44] Le Prell C. G. Gagnon P. M. Bennett D. C. Ohlemiller K. K. (2011b). Nutrient-enhanced diet reduces noise-induced damage to the inner ear and hearing loss. *Transl. Res.* 158 38–53. 10.1016/j.trsl.2011.02.006 21708355 PMC3132794

[B45] Le Prell C. G. Hughes L. Miller J. M. (2007a). Free radical scavengers vitamins A, C, and E plus magnesium reduce noise trauma. *Free Radic. Biol. Med.* 42 1454–1463. 10.1016/j.freeradbiomed.2007.02.008 17395018 PMC1950331

[B46] Le Prell C. G. Yamashita D. Minami S. B. Yamasoba T. Miller J. M. (2007b). Mechanisms of noise-induced hearing loss indicate multiple methods of prevention. *Hear. Res.* 226 22–43. 10.1016/j.heares.2006.10.006 17141991 PMC1995566

[B47] Le Prell C. G. Ojano-Dirain C. Rudnick E. W. Nelson M. A. DeRemer S. J. Prieskorn D. M. (2014). Assessment of nutrient supplement to reduce gentamicin-induced ototoxicity. *J. Assoc. Res. Otolaryngol.* 15 375–393. 10.1007/s10162-014-0448-x 24590390 PMC4010593

[B48] Lee K.-Y. (2013). Pathophysiology of age-related hearing loss (Peripheral and Central). *Korean J. Audiol.* 17 45–49. 10.7874/kja.2013.17.2.45 24653905 PMC3936539

[B49] Li X. Mao X.-B. Hei R.-Y. Zhang Z.-B. Wen L.-T. Zhang P.-Z. (2011). Protective role of hydrogen sulfide against noise-induced cochlear damage: a chronic intracochlear infusion model. *PLoS One* 6:e26728. 10.1371/journal.pone.0026728 22046339 PMC3202565

[B50] Lin F. R. Thorpe R. Gordon-Salant S. Ferrucci L. (2011). Hearing loss prevalence and risk factors among older adults in the United States. *J. Gerontol. A. Biol. Sci. Med. Sci.* 66 582–590. 10.1093/gerona/glr002 21357188 PMC3074958

[B51] Lin Y.-S. Wang C.-H. Chern Y. (2011). Besides Huntington’s disease, does brain-type creatine kinase play a role in other forms of hearing impairment resulting from a common pathological cause? *Aging* 3 657–662. 10.18632/aging.100338 21685512 PMC3164373

[B52] Lin M. T. Beal M. F. (2006). Mitochondrial dysfunction and oxidative stress in neurodegenerative diseases. *Nature* 443 787–795. 10.1038/nature05292 17051205

[B53] López-Otín C. Blasco M. A. Partridge L. Serrano M. Kroemer G. (2013). The Hallmarks of Aging. *Cell* 153 1194–1217. 10.1016/j.cell.2013.05.039 23746838 PMC3836174

[B54] Mathers C. Fat D. M. Boerma J. T. World Health Organization (eds) (2008). *The Global Burden of Disease: 2004 Update.* Geneva: World Health Organization 10.1016/B978-012373960-5.00335-X

[B55] Melgar-Rojas P. Alvarado J. C. Fuentes-Santamaría V. Gabaldón-Ull M. C. Juiz J. M. (2015a). Validation of reference genes for RT–qPCR analysis in noise–induced hearing loss: a study in Wistar rat. *PLoS One* 10:e0138027. 10.1371/journal.pone.0138027 26366995 PMC4569353

[B56] Melgar-Rojas P. Alvarado J. C. Fuentes-Santamaría V. Juiz J. M. (2015b). “Cellular mechanisms of age-related hearing loss,” in *Free Radicals in ENT Pathology*, eds Miller J. M. Le Prell C. G. Rybak L. (Cham: Springer), 305–333. 10.1007/978-3-319-13473-4_15

[B57] Meredith M. A. Stein B. E. (1983). Interactions among converging sensory inputs in the superior colliculus. *Science* 221 389–391. 10.1126/science.6867718 6867718

[B58] Mills D. M. Schmiedt R. A. (2004). Metabolic presbycusis: differential changes in auditory brainstem and otoacoustic emission responses with chronic furosemide application in the gerbil. *JARO J. Assoc. Res. Otolaryngol.* 5 1–10. 10.1007/s10162-003-4004-3 14605922 PMC2538367

[B59] Ohlemiller K. K. (2006). Contributions of mouse models to understanding of age- and noise-related hearing loss. *Brain Res.* 1091 89–102. 10.1016/j.brainres.2006.03.017 16631134

[B60] Ohlemiller K. K. Frisina R. D. (2008). “Age-related hearing loss and its cellular and molecular bases,” in *Auditory Trauma, Protection, and Repair*, eds Schacht J. Popper A. N. Fay R. R. (Boston, MA: Springer), 145–194. 10.1007/978-0-387-72561-1_6

[B61] Overbeck G. W. Church M. W. (1992). Effects of tone burst frequency and intensity on the auditory brainstem response (ABR) from albino and pigmented rats. *Hear. Res.* 59 129–137. 10.1016/0378-5955(92)90110-9 1618705

[B62] Pilati N. Ison M. J. Barker M. Mulheran M. Large C. H. Forsythe I. D. (2012). Mechanisms contributing to central excitability changes during hearing loss. *Proc. Natl. Acad. Sci.* 109 8292–8297. 10.1073/pnas.1116981109 22566618 PMC3361412

[B63] Popelar J. Grecova J. Rybalko N. Syka J. (2008). Comparison of noise-induced changes of auditory brainstem and middle latency response amplitudes in rats. *Hear. Res.* 245 82–91. 10.1016/j.heares.2008.09.002 18812219

[B64] Profant O. Roth J. Bureš Z. Balogová Z. Lišková I. Betka J. (2017). Auditory dysfunction in patients with Huntington’s disease. *Clin. Neurophysiol.* 128 1946–1953. 10.1016/j.clinph.2017.07.403 28826025

[B65] Revuelta M. Santaolalla F. Arteaga O. Alvarez A. Sánchez-del-Rey A. Hilario E. (2017). Recent advances in cochlear hair cell regeneration—A promising opportunity for the treatment of age-related hearing loss. *Ageing Res. Rev.* 36 149–155. 10.1016/j.arr.2017.04.002 28414155

[B66] Schmiedt R. A. (2010). “The physiology of cochlear presbycusis,” in *The Aging Auditory System*, eds Gordon-Salant S. Frisina R. D. Popper A. N. Fay R. R. (New York, NY: Springer), 9–38. 10.1007/978-1-4419-0993-0_2

[B67] Schuknecht H. F. Gacek M. R. (1993). Cochlear pathology in presbycusis. *Ann. Otol. Rhinol. Laryngol.* 102 1–16. 10.1177/00034894931020S101 8420477

[B68] Schuknecht H. F. Watanuki K. Takahashi T. Belal A. A. Kimura R. S. Jones D. D. (1974). Atrophy of the stria vascularis, a common cause for hearing loss. *Laryngoscope* 84 1777–1821. 10.1288/00005537-197410000-00012 4138750

[B69] Seidman M. D. (2000). Effects of dietary restriction and antioxidants on presbyacusis. *Laryngoscope* 110 727–738. 10.1097/00005537-200005000-00003 10807352

[B70] Sendowski I. Holy X. Raffin F. Cazals Y. (2011). “Magnesium and hearing loss,” in *Magnesium in the Central Nervous System [Internet]*, eds Vink R. Nechifor M. (Adelaide: University of Adelaide Press). 29920019

[B71] Sha S.-H. Kanicki A. Halsey K. Wearne K. A. Schacht J. (2012). Antioxidant-enriched diet does not delay the progression of age-related hearing loss. *Neurobiol. Aging* 33 1010.e15–1010.e16. 10.1016/j.neurobiolaging.2011.10.023 22154190 PMC3306450

[B72] Shi X. (2011). Physiopathology of the cochlear microcirculation. *Hear. Res.* 282 10–24. 10.1016/j.heares.2011.08.006 21875658 PMC3608480

[B73] Shone G. Altschuler R. A. Miller J. M. Nuttall A. L. (1991). The effect of noise exposure on the aging ear. *Hear. Res.* 56 173–178. 10.1016/0378-5955(91)90167-8 1769911

[B74] Subramaniam M. Henderson D. Campo P. Spongr V. (1992). The effect of “conditioning” on hearing loss from a high frequency traumatic exposure. *Hear. Res.* 58 57–62. 10.1016/0378-5955(92)90008-B 1559906

[B75] Tavanai E. Mohammadkhani G. (2017). Role of antioxidants in prevention of age-related hearing loss: a review of literature. *Eur. Arch. Otorhinolaryngol.* 274 1821–1834. 10.1007/s00405-016-4378-6 27858145

[B76] Thorne P. R. Nuttall A. L. (1987). Laser Doppler measurements of cochlear blood flow during loud sound exposure in the guinea pig. *Hear. Res.* 27 1–10. 10.1016/0378-5955(87)90021-9 2953704

[B77] Trowe M.-O. Maier H. Schweizer M. Kispert A. (2008). Deafness in mice lacking the T-box transcription factor Tbx18 in otic fibrocytes. *Development* 135 1725–1734. 10.1242/dev.014043 18353863

[B78] Van Eyken E. Van Camp G. Van Laer L. (2007). The complexity of age-related hearing impairment: contributing environmental and genetic factors. *Audiol. Neurotol.* 12 345–358. 10.1159/000106478 17664866

[B79] Vitale C. Marcelli V. Allocca R. Santangelo G. Riccardi P. Erro R. (2012). Hearing impairment in Parkinson’s disease: expanding the nonmotor phenotype. *Mov. Disord.* 27 1530–1535. 10.1002/mds.25149 23032708

[B80] Walling A. D. Dickson G. M. (2012). Hearing loss in older adults. *Am. Fam. Physician* 85 1150–1156. 22962895

[B81] World Health Organization (2017a). *10 Facts on Ageing and Health.* Available at: http://www.who.int/features/factfiles/ageing/en/

[B82] World Health Organization (2017b). *Deafness and Hearing Loss.* Available at: http://www.who.int/mediacentre/factsheets/fs300/en/

[B83] Yamashita D. Jiang H.-Y. Le Prell C. G. Schacht J. Miller J. M. (2005). Post-exposure treatment attenuates noise-induced hearing loss. *Neuroscience* 134 633–642. 10.1016/j.neuroscience.2005.04.015 15961244

[B84] Yamasoba T. Lin F. R. Someya S. Kashio A. Sakamoto T. Kondo K. (2013). Current concepts in age-related hearing loss: Epidemiology and mechanistic pathways. *Hear. Res.* 303 30–38. 10.1016/j.heares.2013.01.021 23422312 PMC3723756

[B85] Yamasoba T. Schacht J. Shoji F. Miller J. M. (1999). Attenuation of cochlear damage from noise trauma by an iron chelator, a free radical scavenger and glial cell line-derived neurotrophic factor in vivo. *Brain Res.* 815 317–325. 10.1016/S0006-8993(98)01100-7 9878807

[B86] Zheng Y. Fan S. Liao W. Fang W. Xiao S. Liu J. (2017). Hearing impairment and risk of Alzheimer’s disease: a meta-analysis of prospective cohort studies. *Neurol. Sci.* 38 233–239. 10.1007/s10072-016-2779-3 27896493

